# Improvement of primary health care of patients with poorly regulated diabetes mellitus type 2 using shared decision-making – the DEBATE trial

**DOI:** 10.1186/1471-2296-13-88

**Published:** 2012-08-22

**Authors:** Eva Drewelow, Anja Wollny, Michael Pentzek, Janine Immecke, Sarah Lambrecht, Stefan Wilm, Iris Schluckebier, Susanne Löscher, Karl Wegscheider, Attila Altiner

**Affiliations:** 1 Rostock University Medical Center, Rostock, Germany; 2 Institute of General Practice, University of Düsseldorf, Düsseldorf, Germany; 3 Institute of General Practice and Family Medicine, University Witten/Herdecke, Herdecke, Germany; 4 Department of Medical Biometry and Epidemiology, University Medical Center Hamburg, Hamburg, Germany

**Keywords:** Diabetes mellitus type 2, Shared decision-making, Primary care, Randomised controlled trial

## Abstract

**Background:**

Since 2004, a national Disease Management Program (DMP) has been implemented in Germany, which includes educational measures aimed at patients with type-2 diabetes (T2D). However, about 15-20% of T2D patients remain in poor metabolic control. Qualitative research shows that one reason for this might be an increasing frustration of general practitioners (GPs) with the management of their poorly regulated T2D patients over time. We aim at approaching this problem by improving the GP-patient-communication and fostering shared decision-making.

**Methods/Design:**

An educative intervention will be tested within a multi-centred cluster-randomized controlled trial (RCT) in Germany. We include 20 GPs in three regions. Each of the 60 GPs will recruit about 13 patients meeting the inclusion criteria (total of 780 patients). GPs allocated to the intervention group will receive a peer-visit from a specifically trained GP-colleague who will motivate them to apply patient-centred communication techniques including patient-centred decision aids. GPs allocated to the control group will not take part in any intervention program, but will provide care as usual to their patients. The primary inclusion criterion for patients at the time of the recruitment is an HbA1c-level of over 8.0. Primary outcome is the change of HbA1c at 6, 12, 18, and 24 months compared to HbA1c at baseline. Secondary outcomes include patient’s participation in the process of shared decision-making and quality of life.

**Discussion:**

If this intervention proves to be effective it may be integrated into the existing Disease Management Program for T2D in Germany.

## Background

In 2004, a national Disease Management Program (DMP) was implemented in Germany. This also includes educational aspects for patients with type-2 diabetes (T2D). However, we know that 15–20% of T2D patients remain in poor metabolic control in terms of their HbA1c level [1]. Poorly regulated HbA1c values are a risk factor for the development of complications like diabetic foot-syndrome, retinopathy, or cardiovascular diseases [2].

Socio-demographical and clinical characteristics alone (e. g. social situation, duration of disease) cannot explain why some T2D patients remain in poor metabolic control. Furthermore, a number of psychosocial factors can influence the quality of blood sugar regulation. These include self-efficacy, personality traits, different coping strategies, various dimensions of stress and social support as well as the relationship to the GP. Also, a flagging involvement of the physician in managing his/her patients has been discussed as another possible reason for constantly poor metabolic control [3-27].

The relationship to and the communication with the GP in the conversation can influence health parameters in general [28]. As Alazri and Neal found, there is a correlation between a higher satisfaction with the GP and better blood sugar values [29].

Interventions focussing on knowledge transfer and understanding the pathophysiology of diabetes seem to have only little impact (e.g. in terms of reaching therapeutic goals) on T2D patients (especially for the non-compliant group) [30,31]. Interventions focussing on the doctor and his practice seem to be promising, in particular when combined with a strong patient-centeredness. Currently, few studies verify these effects on the patient-level (e. g. HbA1c) [32]. Many factors and influencing variables show contradictory effects or were explored in only a few studies with clear methodological limitations. Still, some factors seem to be relevant and promising within the framework of interventions in primary health care.

The continuity of the GP-patient-relationship [33] appears suitable for getting in contact with poorly regulated T2D patients [29]. But sometimes the decreasing involvement and interest of GPs in these patients results in conversations not addressing relevant or conflict-laden issues sufficiently [34].

As shown in the literature, reaching poorly regulated T2D patients and improving their adherence can be fostered by several factors:

a. The GP should be aware of the patient’s illness concepts and keep their significance in mind when dealing with the patient’s disease [35,36].

b. The use of a patient-centred communication behaviour [37,38] (use of evidence-based decision-aids) [39] in the consultation can help reaching the “non-compliant” and complicated patients.

c. Strengthening the perception of the patients’ self-efficacy, “What can I adopt for everyday life and how?” (e.g. good diabetes regulation) [6,7], also seems to be relevant in this context.

Systematic studies over the last 20 years have provided evidence that there is no patent remedy for “successful” interventions to change the behaviour of the GP. But it is indisputable that a successful educative intervention needs to be tailored to the target group in order to implement evidence-based guidelines and patient-centred decision-making [40-43].

Starting point of DEBATE: We know that patients with poor metabolic control and their GPs have often given up on improvement. Due to their gradually built up daily routine they do not see any new possibilities to improve poor blood sugar values [44]. Within this study we want to change the communication behaviour of GPs to foster shared decision-making combined with a lasting patient empowerment. This study’s aim is to verify whether this intervention targeting at GPs is able to:

a. Reduce the HbA1c-value of the included patients by 0,5 compared to the control group.

b. Increase the participation level of the patients in the process of shared-decision making.

## Methods/Design

### Design of the study

An educative intervention will be tested under real conditions in a primary care setting within the framework of a cluster-randomized controlled study. First the participants (GPs and their patients) are recruited and baseline data will be recorded. This is followed by the randomisation of the participating GPs into intervention- and control group. Then data will be recorded at four different measure points (6,12,18,24 months after baseline). GPs allocated to the control group will not take part in any intervention program, but will treat their patients as usual. Patients of those GPs randomized into the intervention group will receive substantial empowerment. It has to be taken into consideration that the recruitment of the GPs and their patients for the study already presents a first intervention, because of the higher attention paid to the problem, by patients and GPs.

### Recruitment

#### GPs

The responsible KV^a^ in every study area provides a list of all GPs taking part in primary care. By sending invitation letters, scientific staff in the three study centres performs different waves of recruitment consecutively until the target number is reached. Each study centre recruits 20 GPs from its catchment area for the study (total = 60).

#### Patients

A computer-generated list will be compiled/created to identify all the eligible patients per practice formally complying with the inclusion criteria. A total number of 780 patients will be needed. For GPs with more than 15 poorly regulated T2D patients on their list, a random sample will be created and the list will also be stratified by gender. Then the GPs consecutively keep inviting their eligible patients until the target number of 13 per practice is reached. Finally, the data will be transferred to the responsible study centre Table 1.

**Table 1:** Inclusion and exclusion criteria

	**Inclusion criteria**	**Exclusion criteria**
On patient-side	Diabetes mellitus type 2	Severe co-morbidity with a life expectancy of less than 24 months
	HbA1c-level over 8.0 at the time of recruitment	
	Ability to give informed consent	
	Sufficient German language skills	
On GP-side	Specialist for general medicine or general practicing internist or GP with KV-admission^a^	

^a^KV-Zulassung = admission of the Association of Statutory Health Insurance Physicians/permission of the panel doctor’s association.

The GPs and patients can end their participation in the study at any time. The treatment of patients who leave the study is guaranteed.

### Sample size/power calculation

Assuming a residual standard deviation of 0.9 a case number of 2x 143 (=286) patients is required to realize a randomized study on the patient-side to show a difference in the HbA1c of 0.5 with a statistical power of 80%. Due to the design of the study (cluster-randomized study with an intervention on the GPs-side) the resulting cluster-effects according to the case number have to be taken into account. The derived factor will be 1.9 assuming an ICC of 0.1 and an average cluster-size of 10. Assuming an average dropout rate of approx. 20%, it is necessary to recruit at least 54 GPs, who treat 13 patients each in order to assure no less than 10 eligible patients per GP [45]. The recruitment of 60 practices is required assuming a practice-drop-out rate of 10%. In order to achieve the calculated sample size (n = 780 patients) the study was designed as a multi-centred study with study centres in Rostock, Düsseldorf and Witten (Institute of General Practice at Rostock University, Institute of General Practice at the Heinrich-Heine University in Düsseldorf, Institute of General Practice and Family Medicine at the University of Witten/Herdecke).

### Randomisation

After the baseline data collection the GPs will be randomized into intervention- and control group.

### Instruments

Further instruments will be used in both groups to measure e.g. the quality of life, the participation within the process of medical decision-making and the empowerment of the patients. The instruments which are utilized are the EQ-5D (measuring the patients health status), the PAID (focussing on the problem areas in Diabetes) the PEF-FB-9 (measuring the congruence of the consultation results and the values or the attitudes of the patients) and the internationally used PACIC-D (measuring aspects of empowerment). At the different points of measurement (T0-T4) a project assistant will contact the patients by phone to disseminate the data with the help of the listed instruments. Additionally, the shortened version of a questionnaire (BÄK questionnaire), developed within a former diabetes project, will be used at baseline (T0) and at the end (T4) of the study to measure how patients live with diabetes [46].

### Data collection/-recording/-transferring

Data collection will take place at different measure points: T0-T4 (6, 12,18 and 24 months after the baseline = T0). The intervention (peer-visit) will take place after the baseline, close to T1 (see intervention).

The following data will be collected at T0 (=baseline):

Concerning the GP: age, gender, time since the practice was established, specialist title, characteristics of the practice, the GP’s marital status and the number of his/her children.

Concerning the patient: age, gender, socio-demographic basic data (educational background, profession, income, marital status, number of children), date of the first diagnosis, pharmacotherapy, initial HbA1c-level, cardiovascular risk prognosis, quality of life (EQ-5D, PAID and BÄK questionnaire), the previous involvement in decision-making processes (PEF-FB-9 and PACIC-D).

The GPs or the GPs’ assistants will collect further details (on): the glycated haemoglobin levels (HbA1c); data needed to determine the cardiovascular risk prognosis (smoking status, familiar predisposition for coronary heart disease (CHD), blood pressure, total cholesterol, HDL cholesterol, LDL cholesterol), pharmacotherapy.

The project’s research assistants will contact the patients by phone and collect all the remaining data (incl. medication taken according to the patient), by means of standardized instruments. The collected data will be recorded in a documentation sheet before being transferred into a saved database.

The data will be collected at the following points in time Table 2:

**Table 2:** Measure points for data collection

	**T0**	**T1**	**T2**	**T3**	**T4**
HbA1c	x	x	x	x	x
EQ-5D and PAID	x		x		x
PEF-FB-9, PACIC-D	x	x	x	x	x
BÄK questionnaire	x				x
Pharmacotherapy	x		x		x
Cardiovascular risk prognosis	x		x		x

### Intervention

The aim of the educative intervention is to enable the participating GPs to identify the agenda and the illness concepts of the patients suffering from poorly regulated diabetes mellitus type 2 and thereby to foster shared-decision making. The focus needs to be more on the patient’s perspective in order to move away from the routine of a more GP-focussed communication without overstraining or unsettling both GPs and patients [47]. For this purpose, a change in the GPs’ communication patterns is necessary. The educational peer-visit presents an integral part of the intervention. Practicing GPs will be specially trained before serving as a peer and doing the educational outreach visit. The peer visit is then supposed to motivate and train the GP in regard to the communicational aspects of the encounter in the GP’s practice, as described in detail in the background section. The GP will also be trained to reflect upon different aspects of the disease and its treatment together with the patient. The training concept follows the Elaboration Likelihood Model [48]. In our preliminary studies we have verified the acceptance of the visits and the positive effects the peer educational outreach visit has on doctor-patient-communication [49].

GPs allocated to intervention will be provided with a computer-based decision-aid, which specifically addresses diabetes and its relation to cardiovascular risk. It is based on the already available tool arriba [50]. The communication tool is meant to be used within the consultation to demonstrate the absolute effects of lifestyle changes and pharmacotherapy in regard to macrovascular event likelihood. [51,52]. Utilizing the decision aid may lead to a more patient-centred communication and enable patients to participate in the decision-making on life-style changes and pharmacotherapy. GPs allocated to intervention will be invited to take part in two group-training sessions to deepen the content of the educational peer visit.

### Outcome measures

Primary endpoint will be the HbA1c-level at the time of the follow-up examinations (T1 = 6 months, T2 = 12 months, T3 = 18 months, T4 = 24 months after baseline). The null hypothesis: the difference of the HbA1c-value of the intervention- and the control group is not higher or lower than 0,5; alternative hypothesis: the HbA1c-value of the intervention group differs at minimum 0,5 from the HbA1c-value of the control group.

Secondary endpoints will include the survey about patients’ quality of life (BÄK questionnaire, EQ-5D, PAID), about the patient-centeredness (PEF-FB-9 and PACIC-D), and about the participation of the patients [53-57]. Data on the calculated cardiovascular risk prognosis and prescribed drugs will also be analyzed.

### Statistical analysis

All analyses will be performed in the ITT-population (intention-to-treat). Instead of the patients, the GPs and their practices will be randomized into clusters. Hence, for the primary analysis, a mixed model with repeated measurements will be adapted at the HbA1c-values of the follow-up. This model includes three levels of hierarchy: GP/practice, patients in the GP’s practice, repeated measurements on the patient-side. Any unstructured covariance matrix will be allowed/admitted for the repeated measurements. First the initial value will be the covariate of the model. An additional covariate on the patient- or GP-level will already be included in the primary analysis, because maldistributions are more typical for cluster-randomized studies than for individually randomized studies. They will be selected right after the patient recruitment has been finalised - considering the baseline values, but without knowing the follow-up values. The coefficient test, comparing the baseline-adjusted final HbA1c-values between the randomized groups, will be performed within the primary analysis. We will use the direct Maximum-Likelihood as the statistical estimation procedure, which results in unbiased estimated values under the missing-at-random-assumption. Alternatively, sensitivity analyses such as last-observation-carried-forward (LOCF) and other worst-case scenarios will be determined. A difference of 0,5% (HbA1c) between the groups will be considered clinically relevant. Depending on their measurement scales the secondary endpoints will be analyzed with analogue models up to the primary endpoint.

### Quality assurance (safety board, gender-specific aspects, interference factors)

The data safety monitoring board (DSMB) will consist of three scientists (one biostatistician and two GPs), who are not associated with the participating institutions performing the study. Based on the interims-analyses of the outcomes the DSMB can vote for a discontinuation of the study.

Some studies refer to gender-specific risk factors and consequences of diabetes [58-60]. To take these into account we will perform analyses stratified by gender. On the level of data analysis we will test whether the intervention reaches the poorly regulated patients with diabetes mellitus type 2 and to what extent. Gender-specific differences might also exist and be relevant for the empowerment-potential.

Confounding variables will primarily be controlled by the randomization. Unevenly allocated characteristics of patients or doctors will be included in the primary assessment model, since the maldistribution of cluster-randomized studies cannot be ignored and therefore needs to be taken into consideration. Potential disturbance variables, which occur after the inclusion will, on the one hand, be minimized by standardizing the study process and handed over essential parts of the documentation to the study assistants. On the other hand, quality assurance measures will be performed within the course of the study. No information regarding the specific hypothesis of the study and the relevance of primary/secondary endpoints will be given to patients and GPs.

### Ethics approval

The study was approved by the Ethic Committee of the University of Rostock on 25^th^ of May 2011.

GPs (after the recruitment procedure) and patients (after the conversation with the GP) will have to sign an informed consent form to participate in the study. Should any patients prematurely cancel the participation in the study they will still receive their medical treatment as usual from the GP.

## Discussion

At this point, no intervention on the improvement of primary health care of patients with poorly regulated diabetes mellitus type 2 has been positively tested in Germany. Most of the GPs continuously use the existing DMP-diabetes module for many of their patients with diabetes. If the designed educative intervention proves to be essentially effective it could be implemented as an important adjunct to the existing DMP module. Our findings will contribute to the optimization of the primary health care routine for the target group. Since our findings are bound to shape the medical conversation concerning content and structure, a higher efficiency can be expected. The position of chronically ill patients as participating and acting subjects will be fostered; this will make a contribution to the improvement of primary health care especially for socially disadvantaged patients by means of a changed, participating communication and increasing competence levels in self-management.

## Endnote

^a^Association of Statutory Health Insurance Physicians

## Competing interests

The authors declare that they have no competing interests.

## Authors’ contributions

AA initiated and designed the study, further development was performed by all authors. The paper was drafted by ED and revised by all authors. All authors read and approved the final manuscript.

## Pre-publication history

The pre-publication history for this paper can be accessed here:

http://www.biomedcentral.com/1471-2296/13/88/prepub
